# Investigating the Formation and Consolidation of Incidentally Learned Trust

**DOI:** 10.1037/xlm0000752

**Published:** 2019-07-29

**Authors:** James W. A. Strachan, Anna á Váli Guttesen, Anika K. Smith, M. Gareth Gaskell, Steven P. Tipper, Scott A. Cairney

**Affiliations:** 1 Department of Cognitive Science, Central European University; 2 Department of Psychology, University of York; 3 Department of Psychology, and York Biomedical Research Institute, University of York; 4 Department of Psychology, University of York; 5 Department of Psychology, and York Biomedical Research Institute, University of York

**Keywords:** trustworthiness, gaze-cueing, sleep, targeted memory reactivation, incidental social learning

## Abstract

People make inferences about the trustworthiness of others based on their observed gaze behavior. Faces that consistently look toward a target location are rated as more trustworthy than those that look away from the target. Representations of trust are important for future interactions; yet little is known about how they are consolidated in long-term memory. Sleep facilitates memory consolidation for incidentally learned information and may therefore support the retention of trust representations. We investigated the consolidation of trust inferences across periods of sleep or wakefulness. In addition, we employed a memory cueing procedure (targeted memory reactivation [TMR]) in a bid to strengthen certain trust memories over others. We observed no difference in the retention of trust inferences following delays of sleep or wakefulness, and there was no effect of TMR in either condition. Interestingly, trust inferences remained stable 1 week after learning, irrespective of the initial postlearning delay. A second experiment showed that this implicit learning occurs despite participants’ being unable to explicitly recall the gaze behavior of specific faces immediately after encoding. Together, these results suggest that gist-like, social inferences are formed at the time of learning without retaining the original episodic memory and thus do not benefit from offline consolidation through replay. We discuss our findings in the context of a novel framework whereby trust judgments reflect an efficient, powerful, and adaptable storage device for social information.

When we observe a person shift their eyes, we experience a powerful shift of our own attention in the direction that they look. This shift of attention results in privileged processing (items are attended, identified, and classified faster and more accurately) of features of the environment that are cued by another’s gaze, compared with those that are not cued (Driver et al., 1999; Friesen & Kingstone, 1998; Frischen, Bayliss, & Tipper, 2007; Langton & Bruce, 1999). This gaze-cueing effect can have downstream effects on cognition, whereby objects that have been the subject of a gaze cue are better remembered (Droulers & Adil, 2015) and liked more (Bayliss, Frischen, Fenske, & Tipper, 2007; Bayliss, Paul, Cannon, & Tipper, 2006; Capozzi, Bayliss, Elena, & Becchio, 2015) than those that were not.

Gaze cues affect not only how we process the environment but also how we process the faces that serve as the sources of gaze-cueing. Bayliss and Tipper (2006) showed participants a set of faces, half of which always looked toward the location of a subsequent target object (valid cues) and half of which always looked away from where the object would appear (invalid cues). They found that when presented with pairs of these faces after the experiment—one valid, one invalid—participants consistently selected the valid faces as the more trustworthy of the two.

This incidental learning—incidental in that participants were never instructed that the faces would provide valid or invalid cues—has been replicated in several studies that have attempted to explore the underlying mechanisms. Emotion has been shown to modulate the gaze-cueing effect, in that validity-contingent trust learning is stronger for smiling faces than neutral or angry ones (Bayliss, Griffiths, & Tipper, 2009; Strachan, Kirkham, Manssuer, & Tipper, 2016). In addition, the effect has been replicated with an economic trust game, showing that the consequences of this learning extend beyond facial trustworthiness judgments: participants were willing to invest in, and even incur real-world costs for, valid-cueing faces over invalid-cueing faces (Rogers et al., 2014). Furthermore, this learned trust appears to be particularly attuned to monitoring the untrustworthiness of invalid faces (Strachan et al., 2016; Strachan & Tipper, 2017).

While the formation and immediate consequences of these incidentally learned trust representations have been studied extensively, there has been comparatively little research addressing the long-term consequences of such learning. Strachan and Tipper (2017) examined this in a series of experiments where they introduced first a short distraction task (approximately 5–6 min watching a series of unrelated videos showing hands picking up objects; Experiments 2 and 3), then an hour-long break away from the lab (Experiment 4) between the gaze-cueing and trustworthiness ratings. They found that the effect was robust to interference, particularly if participants were familiarized with the faces before the experiment. Moreover, evidence of trust learning was observed up to an hour after cueing had ended, suggesting that these incidental representations of trustworthiness are retained for future use. Whether social inferences are retained across longer delays, however, has yet to be examined.

The evidence to date suggests that participants can monitor the gaze behavior of novel faces and make covert social inferences on the basis of this behavior. This incidental learning also appears to undergo a process of retention, thereby affecting social judgments after delays up to one hour, which until now is the longest time period over which this learning has been demonstrated (Strachan & Tipper, 2017). Yet, as previous studies have only looked at single instances of trust learning, it remains unclear how these learned representations are consolidated, change or decay over time. In particular, a key question that arises from an established literature on memory is what role, if any, sleep may play in consolidating these incidentally learned representations of trustworthiness.

## Sleep and Memory Consolidation

Memory decay is reduced across sleep, suggesting that sleep facilitates some forms of consolidation (i.e., the process by which initially labile memory traces become strong and enduring representations; Rasch & Born, 2013). While the majority of studies reporting a memory benefit of sleep have focused on explicitly learned associations (Cairney, Lindsay, Paller, & Gaskell, 2018; Ellenbogen, Hulbert, Stickgold, Dinges, & Thompson-Schill, 2006; Gais, Lucas, & Born, 2006; Payne et al., 2012), other work has demonstrated that implicitly learned information is strengthened across the night (Durrant, Cairney, & Lewis, 2013; Durrant, Taylor, Cairney, & Lewis, 2011). Interestingly, sleep has also been shown to support the development of inferential knowledge regarding hierarchical relationships between separate sets of information (Ellenbogen, Hu, Payne, Titone, & Walker, 2007). Yet, whether social inferences pertaining to facial trustworthiness benefit from overnight memory processing is unknown.

The deepest stage of sleep, known as slow-wave sleep (SWS), has been shown to play a particularly important role in consolidating memories formed via both explicit and implicit learning processes (Durrant et al., 2011, 2013; Gais & Born, 2004; Ngo, Martinetz, Born, & Mölle, 2013; Ngo et al., 2015). According to an influential *active systems* model (Born, Rasch, & Gais, 2006; Diekelmann & Born, 2010; Rasch & Born, 2013), memories are reactivated and thereby strengthened in SWS, promoting long-term storage. An experimental technique known as targeted memory reactivation (TMR) has provided compelling evidence for a role of reactivation in overnight consolidation (for reviews, see Cellini & Capuozzo, 2018; Schouten, Pereira, Tops, & Louzada, 2017). In a typical TMR study, novel information is linked to sounds at encoding; a subset of which are then replayed during SWS in a bid to ‘cue’ the associated memory traces. Memory performance is typically better for cued relative to noncued memories, suggesting that cued representations are selectively reactivated and strengthened during offline periods (Antony, Gobel, O’Hare, Reber, & Paller, 2012; Cousins, El-Deredy, Parkes, Hennies, & Lewis, 2014; Oudiette, Antony, Creery, & Paller, 2013; Rudoy, Voss, Westerberg, & Paller, 2009). Note that TMR effects are typically not observed in wakefulness (Cairney, Guttesen, El Marj, & Staresina, 2018; Rudoy et al., 2009; Schönauer, Geisler, & Gais, 2014; Schreiner & Rasch, 2015), indicating that memory cueing impacts upon mnemonic operations unique to sleep (although see: Oudiette et al., 2013; Tambini, Berners-Lee, & Davachi, 2017). Recent work has furthermore indicated that TMR can be used to stabilize implicitly learned associations (Hu et al., 2015).

In Experiment 1 of the current study, we employed a gaze-cueing procedure to examine how consolidation intervals of sleep or wakefulness influence the decay of validity-contingent trust learning. Using TMR, we furthermore examined whether incidentally learned trust representations for valid-cueing and invalid-cueing faces could be selectively strengthened during sleep or wake. Finally, to probe the persistence of social inferences, we assessed the retention of trust learning following a 1-week delay. Our hypotheses were as follows: (a) incidentally learned representations of trustworthiness would be better retained across sleep relative to wakefulness, (b) the memory benefits of sleep for trust learning would be amplified for representations that were cued via TMR, and (c) trust learning effects would be preserved 1 week after learning.

Another question that we look to address in this study is whether this trust learning is implicit. We describe this effect as incidental learning, as participants are given explicit instructions to ignore the face and to focus on the target objects. As such, any learning about the identities of the faces is the result of tacit processes, as knowing about the trustworthiness of the faces does not help participants complete the task (categorizing objects). Indeed, the only strategic motivation to learn about the faces in this paradigm would be to inhibit misleading gaze cues from untrustworthy identities, but previous research has shown no evidence of such inhibition (Bayliss & Tipper, 2006; Strachan et al., 2016). However, we acknowledge that there is a strong possibility that, while incidental, this learning is not implicit—that is that participants, confronted on every trial with a face that makes valid or invalid gaze cues with 100% reliability, could become aware of the crucial manipulation. As such, in Experiment 2 after gaze cueing is complete, we explain the key manipulation of the study and ask participants to report for each individual face whether it had previously looked toward or away from the target location, to test for explicit awareness of these gaze contingencies.

## Experiment 1

### Method

#### Participants

We recruited 50 participants for Experiment 1. Data for individuals either not returning for the 1-week follow-up session or failing to progress beyond the familiarization stage of the experiment (see Procedure section), were excluded (*N* = 4), leaving 23 participants in the sleep group (11 male; *M*_age_ = 21.25 years) and 23 in the wake group (10 male, *M*_age_ = 20.23 years). Prestudy screening questionnaires indicated that participants had no history of sleep, psychiatric or neurological disorders, were not using any psychologically active medications, had not consumed alcohol or caffeine for 24 hr prior to either experimental session and were nonsmokers. Behavioral exclusion criteria were to remove participants who retained less than 70% of their total data once error removal and reaction time (RT) filters were applied. No participants were removed on this basis. Participants were recruited from the University of York in exchange for £30 or BSc psychology course credit. All participants provided written consent and the research was granted ethical approval by the Research Ethics Committee of the Department of Psychology, University of York.

#### Stimuli

During gaze cueing participants’ task was to categorize objects that appeared on the screen. Target stimuli for this object categorization task were kitchen and garage object images used in Bayliss and Tipper (2006). There were 13 unique objects in each category (kitchen/garage), and these appeared in both horizontal orientations (i.e., if the object had a handle it could point to the left or to the right). All stimuli were colored in blue. In total there were 52 individual images used in the experiment. Face stimuli were taken from the Karolinska Directed Emotional Faces (KDEF) stimulus set (Lundkvist, Flykt, & Öhman, 1998) and included 16 images: eight male and eight female. These faces were initially selected by eye from a figure in the online supplementary material of Oosterhof and Todorov (2008), in which the faces from this set are plotted along six judgment dimensions. The faces used were all taken from the center (1 *SD* from the intersection of all six dimensions) of this plot, so the faces used in our experiments were, compared with the rest of the KDEF set, as close to neutral trait judgments as possible when posing neutral expressions. However, as previous research has shown that trust learning effects are stronger for smiling than neutral faces (Bayliss et al., 2009; Strachan et al., 2016) we used the smiling images for each of the chosen identities.

These faces were split into two sets, which would appear as either 100% valid (always looking toward where the target would appear) or 100% invalid (always looking away) cues in the experiment (counterbalanced across participants). The eyes of each face were manipulated using Adobe Photoshop CS6 to generate faces where the eye gaze was straight ahead, left, or right. Although the original images showed direct gaze, manipulated versions showing direct gaze were generated for the gaze-cueing portion of the experiment so that there would not be a change in sclera texture as a result of the gaze shift. Unaltered images were used for the trustworthiness ratings.

For TMR, each face was also associated with one of two synthetic sounds (A and B, each 1 s in duration) taken from Hu et al. (2015). The sounds can be downloaded from the following link: https://osf.io/q79gv/. An equal number of valid and invalid faces were paired with Sound A and Sound B.

The study was run on an Intel Core i5 PC with a 21.5″ monitor. The experiment was presented using E-Prime 2.0 software with a white background throughout and the resolution set to 1,024 × 768 pixels. Participants sat approximately 60 cm from the display, and during trustworthiness ratings the face stimuli measured 469 × 650 pixels, while during gaze-cueing the face stimuli measured 307 × 461 pixels (these were smaller to account for other images on the screen during gaze-cueing).

#### Design and procedure

**Figure 1 fig1:**
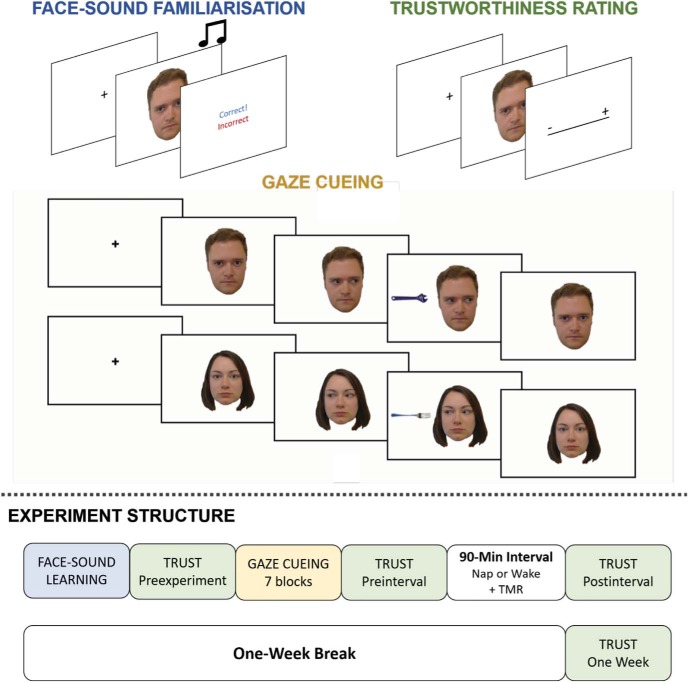
Experimental paradigm. Top part of the image shows the trial structure for the three experimental phases of the study: the face-sound familiarization, trustworthiness ratings, and gaze-cueing. Bottom part of the figure shows full experimental structure with each phase. TMR = targeted memory reactivation. These images are used with permission.

The experimental structure is shown in Figure 1. Experiment 1 consisted of two sessions, which were separated by 1 week. In the first session, participants completed three types of experimental block: face-sound familiarization, trustworthiness ratings, and gaze-cueing (described in detail below). Participants in the sleep condition then took a 90-min nap, while those in the wake condition completed a time-matched filler task. TMR was administered during this 90-min interval (see below). In the second session, participants returned to the lab and completed a set of trustworthiness ratings.[Fig-anchor fig1]

##### Face-sound familiarization

In order to familiarize participants with the faces, which has been shown to lead to more stable trust learning (Strachan & Tipper, 2017), and to ensure that face-sound associations used for TMR were well established, participants completed a familiarization task at the beginning of the experiment.

In this task, two faces were presented on the left and right side of the screen. Each pair of faces consisted of one identity associated with Sound A and one identity associated with Sound B. Participants then heard one of the sounds (A or B) via headphones and were instructed to respond with which of the two faces that sound was associated (the left face or the right face, using the keys Z and M, respectively). The faces would remain until a decision was made, although the sound would only play once. After the participant had made a decision, they were given feedback on whether they were correct or incorrect. As such, while participants started off by guessing, they learned the sound associations over multiple exposures. Participants completed this task as many times as it took for them to go through one full block (each face presented once on the left, once on the right, making 32 trials) without making a single mistake.

##### Trustworthiness ratings

Participants made trustworthiness ratings of all 16 faces used in the experiment. They saw the nonmanipulated original face images showing direct gaze in a random order and were instructed to rate them on trustworthiness by clicking on a linear scale with the mouse. At the beginning of each trial, a calibration screen appeared with the question “How TRUSTWORTHY do you think this person is?” with the word “START” written vertically beneath it. Participants clicked the word “START” to begin the trial, after which the face would appear for 1,000 ms. Then the face disappeared and was replaced with an uninterrupted horizontal line rating scale with “−” and “+” at the left and right end of the line, respectively. Participants were told to click at the point along the line that they thought corresponded to how trustworthy the person was, with more trustworthy ratings closer to the “+” label and less trustworthy ratings closer to the “−” label. The x location of the final mouse position was coded as a point between −100 and +100, with 0 being the absolute center of the line on the screen. We used an uninterrupted line scale with no marks or numbers to reduce the chance of participants explicitly remembering the rating they had previously given particular faces and trying to be consistent with their earlier choices.

Participants completed four trustworthiness rating blocks, during which each face was presented once in a random order, resulting in 16 trials per rating block. The first block followed the face-sound familiarization trials but preceded the gaze-cueing portion of the experiment (*preexperiment rating or baseline*). The second block came immediately after the gaze-cueing but before the 90-min sleep/wake interval (*preinterval rating*). The third block followed the interval (*postinterval rating*). The final block took place 1 week after the original session (*1-week rating*).

##### Gaze-cueing

During the gaze-cueing portion of the experiment, participants were instructed to respond to object images that appeared on the left or right side of the screen after a face had made a gaze shift (i.e., left of right). Participants were explicitly instructed to ignore the face as it was intended to serve as a distractor. They were instead told to focus on deciding whether the object was a kitchen or garage object, using the assigned keys H and the space bar (counterbalanced mapping across participants).

At the beginning of a trial, a fixation cross appeared on the screen for 600 ms. Following fixation, a face appeared on the screen showing direct gaze for 2,500 ms, during which the associated sound would play. Then the face shifted its gaze to either the left or the right. Five-hundred milliseconds after the gaze shift, the object would then appear in the gaze-cued (valid trial) or gaze-uncued (invalid trial) location. The target object remained on screen for 3000ms or until the participant’s kitchen/garage response was logged. The face then shifted back to direct gaze for 1,000 ms, followed by a 1,000-ms feedback screen which showed “XX” in red for trials where an error was committed (i.e., incorrect kitchen/garage decision). Feedback was only provided on error trials in an effort to reduce posterror slowing (Compton, Heaton, & Ozer, 2017). There was a 500-ms blank display interval between trials.

In total there were seven blocks with 32 trials each, with each face appearing twice in each block, once gazing left and once right. As such, each face appeared 14 times in total throughout the experiment, always producing either valid or invalid cues (depending on the identity).

##### Sleep and wake delays

Participants in the sleep condition were left to nap in a laboratory bedroom for 90 min while their brain activity was monitored with polysomnography (PSG). An Embla N7000 PSG system with RemLogic 3.4 software was used to monitor sleep. After the scalp was cleaned with NuPrep exfoliating agent (Weaver and Company, Aurora, CO, USA), gold plated electrodes were attached using EC2 electrode cream (Grass Technologies, West Warwick, RI, USA). EEG scalp electrodes were attached according to the international 10–20 system at six locations: frontal (F3, F4), central (C3, C4), and occipital (O1, O2), and each was referenced to the contralateral mastoid. Left and right electrooculography electrodes were attached, as were electromyography (EMG) electrodes at the mentalis and submentalis bilaterally, and a ground electrode was attached to the forehead. Each electrode had a connection impedance of <5 kΩ. All online signals were digitally sampled at 200 Hz. Sleep scoring was carried out in accordance with the criteria of the American Academy of Sleep Medicine (Berry, Brooks, Gamaldo, Harding, & Vaughn, 2012).

TMR was initiated when participants were in the N2/SWS transition. The sound (A or B) was played continuously with a randomized interstimulus interval between 3.5 s and 6.5 s. TMR continued for as long as participants were in SWS, but immediately paused if they showed signs of microarousal, awakening or transition into another sleep stage. TMR was restarted if participants reentered SWS.

To habituate participants to auditory stimulation during sleep, and thus reduce the risk of arousals or awakenings during sound replay, low-intensity Brown noise was played into the bedroom for the entirety of the nap phase. The overall sound intensity (TMR cues + background noise) was ∼50 dB.

Participants in the wake group played the online game Bubbleshooter (http://shooter-bubble.com) for the first 30 min of the interval. After this, they completed a working memory task. On each trial, a series of random letters were presented, one after another, in-between valid or invalid sentences, with the series length varying from two to seven letters. Participants were required to retain the series of letters in working memory while making judgments on the validity of the sentences that appeared between each letter, and then report the full series of letters at the end of the trial. At the same time as completing this task, the TMR sound was presented as described above. This approach ensured that participants were sufficiently distracted from the sounds (Cairney, Guttesen et al., 2018), and, thus, would be unlikely to actively retrieve the associated faces. After completing the working memory task, participants played Bubbleshooter again for the remaining 30 min of the interval.

#### Data analysis

Data of interest were RTs and accuracy rates during gaze cueing, and reported trustworthiness ratings at each stage of the experiment.

##### Gaze-cueing analysis

For the analysis of gaze cueing, all trials were initially filtered such that RTs below 250 ms (indicating anticipatory responses that were too fast to process the stimulus) and above 3,000 ms (responses made after the critical window where the target was present) were marked as incorrect. This amounted to a total of 0.34% of trials across all participants. For accuracy analysis (i.e., proportion of correct kitchen/garage object responses) we then averaged across trials for each participant in each condition to create a total proportion correct. These were then analyzed using the *ez* package in R.

We then filtered the data such that only correct trials were included. For all trial exclusions, we established a priori that any participants who retained less than 70% of the total number of trials following filtering (indicating too many anticipatory, delayed, or erroneous responses) would be removed from all analyses. No participants were excluded on this basis. RTs were then analyzed using the *ez* package in R. The total proportion of excluded trials was 2.83%.

##### Trust ratings

Trust ratings were recorded in four sessions: *preexperiment* (before gaze cueing), *preinterval* (before the 90-min sleep/wake interval), *postinterval* (immediately following interval), and *1 week*. in each session, ratings were between −100 and +100 for each identity used in the experiment.

For the first run of analysis we examined whether incidental trust learning effects were replicated. We included session and validity as independent factors in a repeated measures ANOVA. However, because we were principally interested in the change in learning between later sessions, we transformed the data into trust change scores to remove the influence of baseline ratings from this analysis. For each participant, we first generated mean trustworthiness ratings for each session, for each level of face validity. Using the preexperiment mean ratings as baseline, at each subsequent session we calculated the change in trust from baseline for valid and invalid types of face separately. We submitted these change scores to a 2 × 3 (Validity: Valid, Invalid × Session; Preinterval, Postinterval, 1 Week) repeated measures ANOVA to see if there was decay in trust learning over time. For the three sessions’ change scores we ran separate follow-up Bonferroni-corrected *t* tests to test whether trust learning was significant at each time point.

When analyzing the data in terms of the effect of sleep, we were particularly interested in how sleep (and TMR) affected the magnitude of learned trustworthiness judgments, both between the pre- and postinterval sessions, and across the week-long interval (relative to the postinterval session).

To address this question, we first calculated a trust learning index at each time point (session) by subtracting invalid change scores from valid changes scores (as such, a greater value indicated a more extreme distinction in trust scores based on the gaze validity of the faces). Next, we calculated a *trust change index* for the postinterval session (*t* = 3) and the 1-week session (*t* = 4) using Formula 1 separately for TMR-cued and noncued faces (TMR-on and TMR-off) in the sleep and wake groups. These indices were then compared in separate (one per session of interest) 2 × 2 mixed ANOVAs with group (sleep/wake) as a between-subjects factor and TMR (on/off) as a within-subjects factor.

$$\left(V_{t}-\;I_{t})-(V_{t-1}-I_{t-1}\right)=\text{trust change index}$$

##### Formula 1

Formula to calculate a trust change index for the third and fourth trust rating sessions. *V* and *I* indicate baseline-adjusted trustworthiness ratings to valid and invalid faces, respectively. *t* refers to the session of interest (*t* = 3: postinterval session; *t* = 4: 1-week session).

All trust ratings were again analyzed using the *ez* package in R. Wherever Mauchly’s assumption of sphericity was violated we used a Greenhouse Geisser correction of the degrees of freedom.

Null results that were considered theoretically important were followed up with Bayesian analyses to evaluate the evidence in support of the null. This Bayesian analysis involved calculating a Bayes factor indicating the probability of the observed data under the null hypothesis (H0) relative to the alternative hypothesis (H1; BF_01_). This allows for a clearer interpretation of the data. For example, BF_01_ = 3 indicates that the data are three times more likely under H0 than H1. Bayesian information criterion probabilities (pBIC) were also calculated as these provide a graded level of evidence regarding which model (H1 or H0) is more strongly supported, given the data. These values are calculated using an approach that applies simple transformations to the sum of squares of the frequentist ANOVA, as outlined in Masson (2011).

### Results

#### Gaze-cueing

##### Object accuracy

Percentage of accurate kitchen/garage object responses are shown for each condition in Table 1. Error rates were analyzed using mixed ANOVA with accuracy (proportion of correct trials) as a dependent variable and with validity (valid/invalid) and group (sleep/wake) as factors. Accuracy was slightly higher in the sleep group than in the wake group, but the effect of condition was not statistically significant, *F*(1, 44) = 3.70, *p* = .061, $\eta_{p}^{2}$ = 0.08. There was no main effect of validity, *F*(1, 44) = 0.02, *p* = .898, $\eta_{p}^{2}$ = 0.00 and no interaction between the two factors, *F*(1, 44) = 2.40, *p* = .128, $\eta_{p}^{2}$ = 0.05.[Table-anchor tbl1]

##### Reaction times

**Figure 2 fig2:**
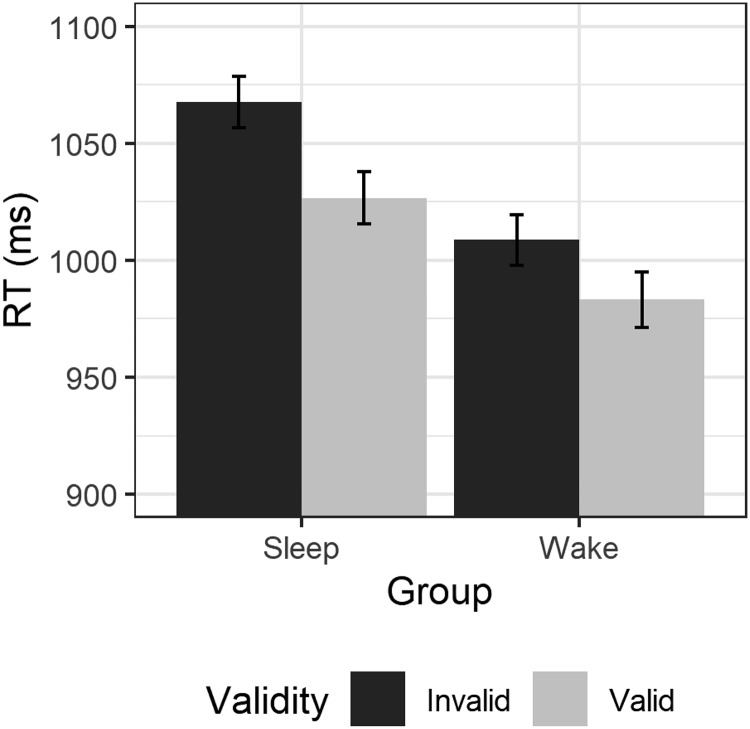
Average RTs to valid (light gray) and invalid trials (dark gray bars) for those participants in the sleep (left) and wake conditions (right). Error bars show ±1 within-subjects standard error.

Average RTs are shown in Figure 2. RTs were analyzed in a 2 × 2 mixed ANOVA with validity (valid/invalid) as a within-subjects factor and group (sleep/wake) as a between-subjects factor. The ANOVA revealed a main effect of validity, reflecting a classic gaze-cueing effect such that RTs were faster to valid than invalid trials, *F*(1, 44) = 17.45, *p* < .001, $\eta_{p}^{2}$ = 0.28. Although RTs were slightly faster in the wake condition than in the sleep condition, there was no main effect of group, *F*(1, 44) = 0.57, *p* = .456, $\eta_{p}^{2}$ = 0.01, and there was no interaction between factors, *F*(1, 44) = 0.72, *p* = .399, $\eta_{p}^{2}$ = 0.02.[Fig-anchor fig2]

Previous research has indicated that people form trust representations online during gaze-cueing (Manssuer, Pawling, Hayes, & Tipper, 2016; Manssuer, Roberts, & Tipper, 2015), but that this learning does not affect individuals’ responses to misleading cues (Strachan et al., 2016). That is, although people learn that certain faces are untrustworthy, they do not use this strategically to inhibit the automatic reorienting of attention to the cued side of space. We ran an exploratory repeated measures ANOVA analysis on RTs with validity and block (1–7) as factors, and while we found significant effects of validity, *F*(1, 45) = 19.01, *p* < .001, $\eta_{p}^{2}$ = 0.30 and block, *F*(9.95, 447.92) = 77.51, *p* < .001, $\eta_{p}^{2}$ = 0.63, there was no interaction between the two, *F*(8.17, 367.82) = 1.56, *p* = .182, $\eta_{p}^{2}$ = 0.03. See Supplementary Figure 1 for RTs broken down by validity and block.

#### Trustworthiness ratings

##### Trust learning

**Figure 3 fig3:**
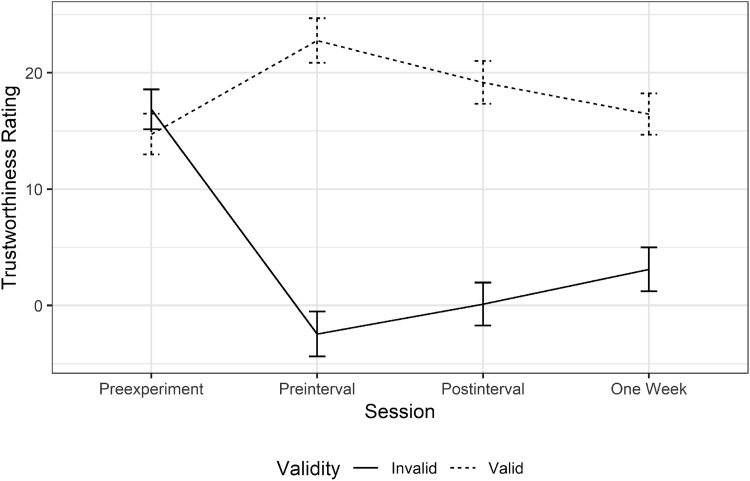
Average trustworthiness ratings assigned to faces that appeared in valid (dashed lines) and invalid trials (solid lines) during gaze cueing in the four different sessions. All error bars show ±1 within-subjects standard error.

All trust ratings across the sleep and wake groups are shown in Figure 3. For the first run of analysis, data were collapsed across groups in a 2 (Validity) × 4 (Session) repeated-measures ANOVA. This analysis found a main effect of Session (Greenhouse-Geisser [GG] corrected: *F*(2.35, 105.76) = 8.86, *p* < .001, $\eta_{p}^{2}$ = 0.16), and a main effect of validity, *F*(1, 45) = 13.48, *p* = .001, $\eta_{p}^{2}$ = 0.23. There was also a significant interaction (GG corrected: *F*(1.56, 70.19) = 15.22, *p* < .001, $\eta_{p}^{2}$ = 0.25).[Fig-anchor fig3]

This analysis replicates and extends previous findings of trust learning effects (Manssuer et al., 2015, 2016; Strachan, Kirkham, Manssuer, Over, & Tipper, 2017; Strachan et al., 2016; Strachan & Tipper, 2017). There was no difference between valid and invalid faces at the first rating, while at each subsequent time point participants rated valid faces as more trustworthy than invalid faces.

Next, we examined whether there was a change in the magnitude of this learned trust over time; either exaggeration or decay. We calculated trustworthiness change scores for each of the three postlearning sessions relative to the preexperiment baseline (preinterval, postinterval, and 1 week) and subjected these to a 2 × 3 (Validity × Session) ANOVA.^1^ This did not reveal a significant main effect of session (GG corrected: *F*(1.57, 70.46) = 0.08, *p* = .923, $\eta_{p}^{2}$ = 0.00). However, there was a significant main effect of validity, *F*(1, 45) = 18.49, *p* < .001, $\eta_{p}^{2}$ = 0.29. Crucially, there was also an interaction of the two factors, *F*(2, 90) = 8.21, *p* = .001, $\eta_{p}^{2}$ = 0.15, indicating that there was significant change in the effect over time. By looking at the results in Figure 3, we can see that this change is a decay in the magnitude of the effect.

To explore this interaction further we compared these values at each time point in separate *t* tests (Bonferroni corrected α = .017) and found that differences in learned trust ratings were significant immediately following trust learning, *t*(45) = 4.54, 95% CI [15.22, 39.45], *p* < .001, *d* = 0.67, and following a 90-min interval (sleep or waking distraction; *t*(45) = 3.87, 95% CI [10.15, 32.17], *p* < .001, *d* = 0.57). Interestingly, while differences between valid and invalid ratings were smaller 1 week later, indicating that these trust representations did decay over time, there was still evidence of significant trust learning, *t*(45) = 3.84, 95% CI [7.34, 23.58], *p* < .001, *d* = 0.57. This is the longest retention of this effect that has been experimentally demonstrated.

##### Sleep and targeted memory reactivation

**Figure 4 fig4:**
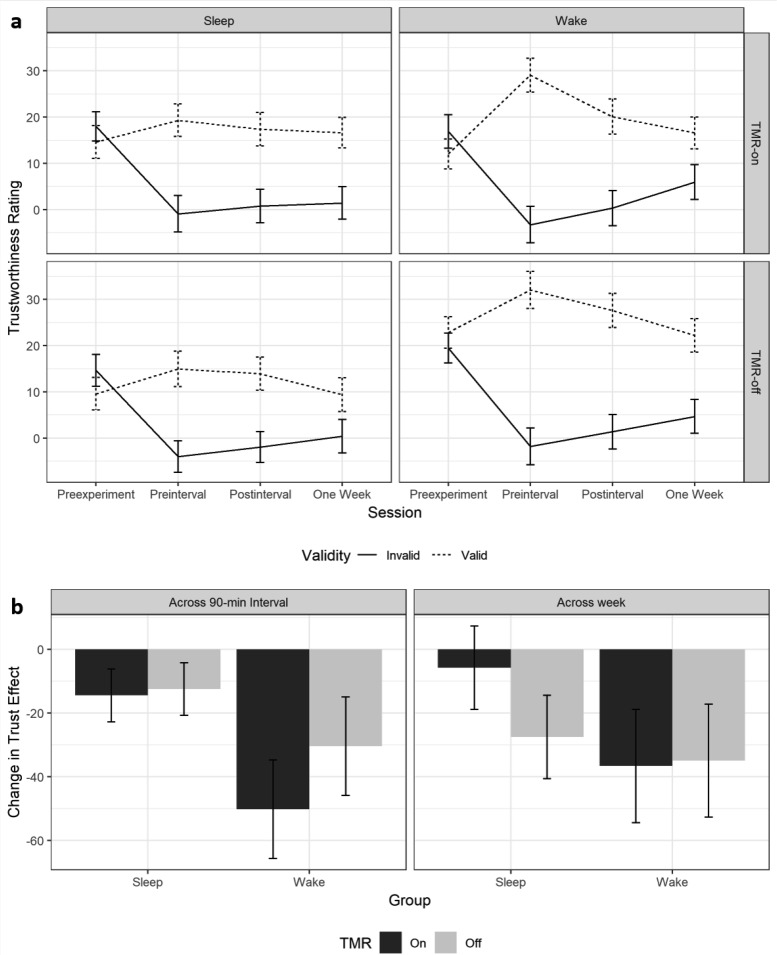
a. Timecourse of average trustworthiness ratings assigned to faces that appeared in valid (dashed lines) and invalid trials (solid lines) during gaze cueing in the four different sessions in response to targeted memory reactivation (TMR)-on (top row) and TMR-off faces (bottom row) in the sleep (left plots) and wake groups (right plots). b. Change in incidentally learned trust effect (trust change index) over the course of the 90-min interval relative to the preinterval differences (left plot) and over the week break relative to the postinterval differences (right plot) for participants in the sleep and wake groups (left and right bars, respectively) in response to faces which were subjected to TMR (dark gray bars) or were not (light gray bars). Error bars show ±1 within-subjects standard error.

We next examined the effects of sleep and TMR on the consolidation of incidental trust learning over time. The raw trustworthiness ratings for each condition are shown in Figure 4a. To analyze these, we generated the same change scores from baseline described in the previous analysis separately for TMR-on and TMR-off faces in the sleep and wake groups. We then subtracted the change scores for invalid faces from the change scores for valid faces to calculate a trust learning index. This difference score provides a metric for the magnitude of the incidental trust effect at any given time, in response to TMR-on and TMR-off faces in the sleep and wake groups. We were interested in the effects of sleep and TMR at two time points: immediately after the sleep/wake interval and 1 week later, as recent work has shown that TMR can lead to behavioral effects that do not emerge immediately (Cairney, Guttesen et al., 2018; Simon, Gómez, & Nadel, 2018). These trust change scores (calculated using Formula 1 and shown in Figure 4b) allowed us to observe any change in the total trust effect between these sessions as a function of group and TMR. Negative values indicate decay over time. Thus, less negative change indicates a consolidation benefit.[Fig-anchor fig4]

We subjected these trust change indices to separate 2 × 2 mixed ANOVAs, with group (sleep/wake) as a between-subjects factor and TMR (on/off) as a within-subjects factor. Immediately following the sleep/wake interval, there was no main effect of group, *F*(1, 42) = 1.77, *p* = .191, $\eta_{p}^{2}$ = 0.04, nor of TMR, *F*(1, 42) = 0.75, *p* = .391, $\eta_{p}^{2}$ = 0.02 and no interaction, *F*(1, 42) = 0.54, *p* = .466, $\eta_{p}^{2}$ = 0.01. These findings suggest that the consolidation of trust learning is unaffected by sleep and/or TMR. A Bayesian model selection approach was used to evaluate evidence in support of these null hypotheses (Bayesian Information Criterion [BIC], Masson, 2011, see Method section). These analyses provided moderate support for the null hypotheses that sleep and TMR did not affect the consolidation of incidentally learned trust (null model vs. group main effect, BF_01_ = 2.68, pBIC(H1|D) = 0.27, pBIC(H0|D) = 0.73; null model versus TMR main effect, BF_01_ = 4.36, pBIC(H1|D) = 0.19, pBIC(H0|D) = 0.81; null model versus group*TMR interaction, BF_01_ = 5.01, pBIC(H1|D) = 0.17, pBIC(H0|D) = 0.83). For each of these contrasts, the relative probability of the observed data under alternative hypothesis is smaller than would be expected even for a weak effect (pBIC(H1|D) = 0.5–0.75), and evidence consistently favors the null.

One week later, there was similarly no effect of group, *F*(1, 42) = 0.63, *p* = .432, $\eta_{p}^{2}$ = 0.01 or TMR, *F*(1, 42) = 0.47, *p* = .496, $\eta_{p}^{2}$ = 0.01 and no interaction between the two factors, *F*(1, 42) = 0.58, *p* = .450, $\eta_{p}^{2}$ = 0.01. A Bayesian approach was again used to assess the evidence in support of each null hypothesis. As above, these analyses provided moderate support for the null effects of sleep and TMR (null model vs. group main effect, BF_01_ = 4.78, pBIC(H1|D) = 0.17, pBIC(H0|D) = 0.83; null model versus TMR main effect, BF_01_ = 5.32, pBIC(H1|D) = 0.16, pBIC(H0|D) = 0.84; null model versus group*TMR interaction, BF_01_ = 4.90, pBIC(H1|D) = 0.17, pBIC(H0|D) = 0.83). Note that, in the sleep group, the magnitude of the trust learning effect (across TMR conditions) at the postinterval or 1-week sessions was not predicted by time spent in any stage of sleep or total sleep time (*p* > .05).^2^

### Discussion

Experiment 1 replicated previous studies that demonstrate incidental trust learning from gaze cues. Furthermore, for the first time, we showed that this learning was stable up to one week after initial encoding. However, our results indicate that there is no effect of sleep on the consolidation of incidentally learned trust representations. There was also no effect of TMR on learning in either the sleep or wake groups. A Bayesian model selection approach provided confirmatory evidence for these null effects.

This is the longest retention of incidentally learned trust from gaze cues that has been demonstrated to date. The fact that participants retained memory for trust associations after a week replicates and extends previous findings indicating that incidental trust learning is a long-term mnemonic effect (Strachan & Tipper, 2017), rather than reflecting only online monitoring of gaze contingencies. It is also important to note that there was evidence of decay in trust learning—differences between trustworthiness ratings of valid and invalid identities were smaller following a week-long gap than they were initially following gaze-cueing. As this is the first study to measure trust learning at multiple time points we cannot draw conclusions from this decay, but it could be an avenue for future research to investigate this further. For example, this decay in trust learning may be mitigated by contextual or relevance factors—it may not be worth the mental resources to indefinitely preserve the memory of an unfamiliar face that appeared on a screen, unless there is reason to expect that such a representation may be relevant in the longer term. Investigating the factors affecting trust decay and studying how decay in trust learning compares with decay in other types of memory systems, raises some interesting questions.

The finding that sleep immediately following trust learning did not lead to stronger or more durable trust representations is surprising; previous work suggests that sleep not only facilitates consolidation across a range of memory domains, but also supports the development of inferential knowledge (Ellenbogen et al., 2007). Based on these earlier findings, Lewis and Durrant (2011) proposed a model of overnight consolidation where memory reactivation in sleep serves to abstract gist information from newly formed memory traces. During reactivation, overlapping memory features are thought to undergo the greatest strengthening, and become the foundations of cognitive schemata. The idiosyncratic and nonoverlapping features of individual episodes are consequently lost as they are not subject to such selective strengthening.

However, in the current experiment individual episodes (trials) had highly relevant overlapping features (each face always looked toward or away from the object). When participants are exposed to multiple similar, low-arousal instances of valid or invalid gaze behavior, this model suggests that they do not remember these episodes discretely—a costly and inefficient strategy—but instead during sleep they would aggregate these episodes and extract some relevant metadata from the encounter (e.g., this person’s behavior was untrustworthy) that is then passed to a gist or summary representation of that person’s identity. Nonoverlapping data, such as the object that participants responded to, or whether the face looked left or right on a given trial, would be lost. Such a storage mechanism would be efficient, stable, and—for the purposes of the current experimental task—sufficient. Yet, there was no evidence of such abstraction during sleep.

An explanation for why we did not observe stronger trust effects following a nap could be that a stable, efficient trustworthiness representation (or gist) is already stored after gaze cueing, and so there is no additional benefit of sleep. That is, rather than aggregating and extracting gist information during sleep, participants construct a representation of particular individuals in situ, passing on the relevant impressions of trustworthiness and discarding irrelevant idiosyncratic information during memory formation (gaze cueing). This representation would essentially create a ceiling effect—the simple classification of faces as trustworthy or untrustworthy creates much lower load demands than specifically tracking individual behavior, and this ceiling effect means that sleep has no additional benefit. This view is consistent with the notion of generalization arising from integrative encoding, whereby overlapping events are integrated into a blended representation across multiple encoding episodes (Shohamy & Wagner, 2008; although see also retrieval-based models of emergent generalization: Banino, Koster, Hassabis, & Kumaran, 2016; Kumaran & McClelland, 2012). Furthermore, given the prompt feedback provided on each gaze-cueing trial, the resultant trust representations might have already depended on striatal memory systems to a greater extent than those in medial temporal areas (Foerde, Race, Verfaellie, & Shohamy, 2013; Foerde & Shohamy, 2011), potentially diminishing a memory benefit of sleep (Durrant et al., 2013; Rasch & Born, 2013). More work is needed to fully uncover the mnemonic impacts of sleep.

Because sleep had no impact on the retention of incidentally learned trust representations, it is perhaps not surprising that TMR also had no impact on behavior. It should be noted, however, that TMR experiments typically probe retrieval of the same information encountered at encoding (e.g., the recall of adjective-image pairs encoded in the presleep training phase; Cairney, Guttesen et al., 2018). The current study required the retrieval of trustworthiness inferences based on gaze cueing behavior at encoding, which are highly distinct. Whether any observable benefit of sleep for inferential learning (such as that seen for hierarchical relationships; Ellenbogen et al., 2007) would be amplified by TMR is therefore an open question. Interestingly, recent work has suggested that TMR abolishes rather than enhances sleep-related generalization (Hennies, Lambon Ralph, Durrant, Cousins, & Lewis, 2017).

If participants had extracted gist information before sleep (i.e., during gaze-cueing) as part of a cognitive cost-saving mechanism, then they should not have retained source memory of individual episodes. It would follow that, even immediately following gaze-cueing, participants would not be able to explicitly recall the gaze behavior of individual faces, as the source memory of the individual episodes used to generate the gist is not retained beyond its integration into the trust representation. It has never been explicitly tested with the current paradigm whether participants can actually remember the gaze behavior of individual faces. Bayliss et al. (2009) did include a manipulation check to show that, even when debriefed, participants could not recall which faces had looked at the target throughout a gaze-cueing experiment. However, Bayliss et al. (2009) did not include a trustworthiness rating at the beginning of the study. This baseline rating may cue participants to more closely consider the trustworthiness of faces during the experiment. It is important to know whether participants are explicitly aware of how gaze cues inform their trustworthiness judgments in the current study, or whether they learn to trust or distrust faces without being able to explicitly report the behavior of those faces. An implicit learning effect would be consistent with our view that abstraction of trust representations does not require sleep.

In order to address this question we report the results of a second experiment that was run independently of Experiment 1, which we feel can inform the current study. Participants completed a trustworthiness preexperiment rating and then a gaze-cueing procedure similar to our previous work (Strachan et al., 2016, 2017; Strachan & Tipper, 2017). However, instead of a final trustworthiness rating, participants were fully debriefed about the behavior of the faces and asked to explicitly report which way each face had looked during the experiment; that is, whether consistently toward or away from targets.

## Experiment 2

### Method

In this experiment, participants completed the same gaze-cueing task with valid and invalid faces as in Experiment 1, but rather than rate faces for trustworthiness at the end of the experiment, they were asked to explicitly recall the gaze behavior of each face.

#### Participants

Thirty-one participants volunteered for this study in return for a mixture of course credit and payment (£3 for 30 min). One participant’s data was not collected due to a runtime error, so the total number available for analysis was 30 (five male, gender information not collected for three; *M*_age_ = 21.79 years).

#### Stimuli, design, and procedure

Experiment 2 was broadly similar to Experiment 1, in that the gaze-cueing procedure included the same face identities providing 100% valid or invalid gaze cues throughout the experiment. However, as Experiment 2 was conceived and conducted independently of Experiment 1 there were some methodological differences. These are outlined in detail below.

First, because this was not a TMR experiment, the face-sound association task from Experiment 1 was not included in Experiment 2. Participants still rated all faces for trustworthiness at the beginning of the experiment, but we did not include a trustworthiness rating at the end of the experiment alongside the awareness check. This was because the two measures would likely contaminate each other—if participants deduced that the experiment related to trust and gaze behavior, they may use the trustworthiness ratings they give to faces to inform guesses of gaze behavior even in the absence of true explicit memories. Although this means that it is not possible to confirm that these participants have indeed acquired these trust representations, we test explicit awareness here at the same point in the experiment (immediately after gaze cueing) that several previous studies have shown that participants can and do form robust and reliable trust learning effects using similar paradigms (Manssuer et al., 2015, 2016; Strachan et al., 2016, 2017; Strachan & Tipper, 2017).

#### Stimuli

Faces used in the experiment were the same identities as in Experiment 1, but where Experiment 1 used smiling faces, Experiment 2 used neutral expressions as stimuli throughout.

##### Gaze cueing

Participants completed five blocks of gaze-cueing rather than the even blocks in Experiment 1.^3^ Because no sounds were presented at gaze cueing in Experiment 2, the trial timings were also slightly different: there was again a 600 ms fixation, followed by the face showing direct gaze for 1,500 ms. The face then shifted gaze left or right for 500 ms before the target object appeared. The target object then remained for 2,500 ms, and an error tone would play after this window if the participant had failed to respond or had miscategorized the object (i.e., incorrect garage/kitchen object response). The face then shifted back to direct gaze for 1,000 ms, followed by a blank screen for 500 ms.

##### Awareness check

The crucial feature of Experiment 2 was that following gaze cueing participants did not complete another set of trustworthiness judgments. Instead, an instruction screen appeared and explained the crucial experimental manipulation during gaze-cueing: that each face during the experiment had either always looked toward or away from where the object was about to appear. They were then told that the next procedure involved them having to recall whether each face had looked toward or away from the object.

For each trial in the gaze awareness procedure, a face appeared in the center of the screen with the question, “Did this face look TOWARDS or AWAY from the object?” and response key reminders on either side of the screen. Participants were instructed to press Z if they felt the face had looked toward where the object had been about to appear, M if they felt the face had looked away, and the SPACE bar if they could not remember—across all participants, reports of not knowing occurred in 14.42% of trials, although this varied across participants (*SD* = 16.81%). Faces were shown once in a randomized order.

As such, the overall structure of Experiment 2 was that participants completed preexperiment trustworthiness judgments of all faces, then did five blocks of the gaze-cueing task, followed by the gaze awareness procedure. Participants completed only one session and did not return to the lab once the awareness check was completed.

#### Data analysis

RTs and accuracy were preprocessed as in Experiment 1, with the difference that the upper RT filter was adjusted from 3,000 ms to 2,500 ms to reflect the new trial timings. As the only independent variable in this experiment was validity, RTs and accuracy rates were compared in separate paired-samples *t* tests. Errors were low (3.21% of trials) and RT outliers were rare (<0.4% of trials), and no participants were excluded on either basis.

For gaze awareness results, participants’ data was marked as incorrect if the participant chose the wrong cueing behavior or if they pressed the SPACE bar to indicate that they did not know. As there were 16 faces, each participant could score a total number correct out of 16. Chance level (50% correct) was eight out of 16, and binomial tests indicated that 12 was the threshold at which recall could be considered significantly above chance. As such, participants scoring 12/16 correct or above were considered aware of the manipulation and face cueing behavior, while those scoring below this were considered naïve to the manipulation. We also include the results of a Bayesian binomial test on choice outcomes to evaluate evidence for or against the null hypothesis, calculated using JASP v.0.9.0.1. BIC values were calculated using the Bayes factor value.

We categorized participants on this individual basis, but we also calculated the total number of successes across all participants and tested whether these differed significantly from chance accuracy using a binomial test. This enabled us to determine whether evidence of awareness emerged at the population level where it might not at the individual level.

### Results

#### Gaze cueing

**Figure 5 fig5:**
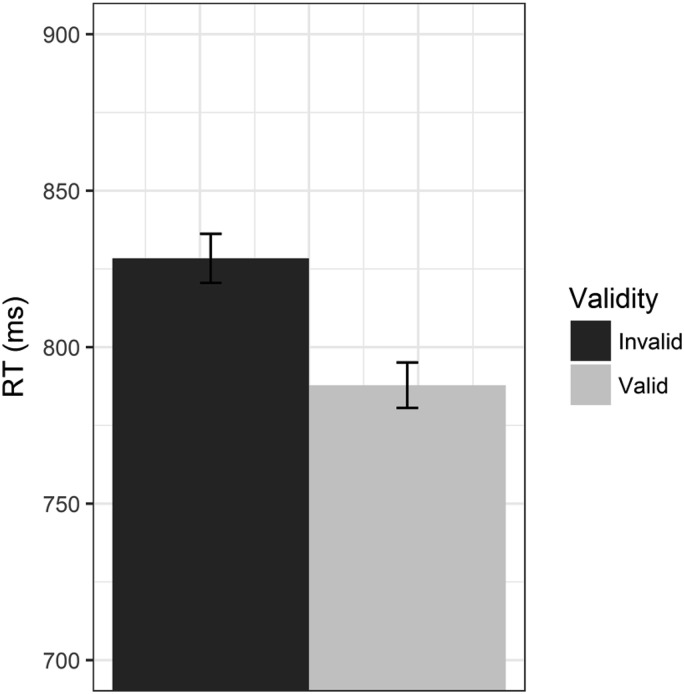
Average RTs to valid (light gray) and invalid trials (dark gray bars) for Experiment 2. Error bars show ±1 within-subjects standard error.

Error rates in Experiment 2 were low, and although accuracy was slightly higher in response to valid gaze cues (*M* = 97.29%, *SE* = 2.96) than invalid (*M* = 96.29%, *SE* = 3.45), this difference was a nonsignificant trend, *t*(29) = 1.97, 95% CI [−0.00, 0.02], *p* = .058, *d* = 0.73. On the other hand, RTs (see Figure 5) were significantly faster to valid trials than invalid trials, *t*(29) = 3.87, 95% CI [60.48, 18.64], *p* < .001, *d* = 1.44, indicating a classic gaze-cueing effect.[Fig-anchor fig5]

#### Awareness

**Figure 6 fig6:**
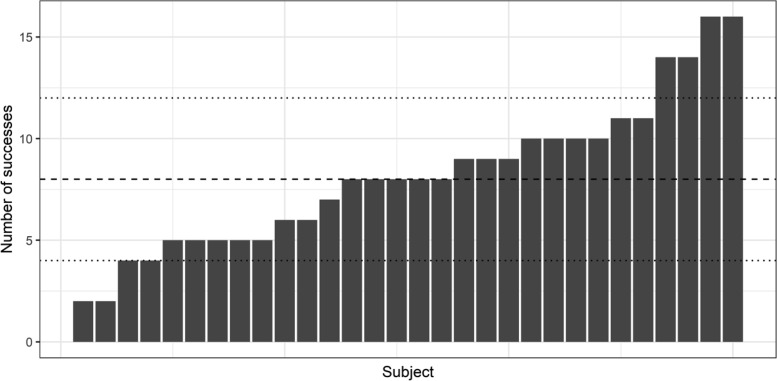
Total number of correct answers (out of possible 16) for each individual participant in Experiment 2 gaze awareness check, arranged from poorest to best performance. Taller bars indicate greater accuracy when asked to report the gaze behavior of individual faces. Dashed line indicates 50% (chance) performance. Dotted lines indicate thresholds above (or below) which binomial tests indicated significant above-chance (or below-chance) performance.

The results of the awareness check are shown in Figure 6. Out of 30 participants, only four (13.3%) reached the previously defined threshold for significant above-chance accuracy (more than 12 out of 16 correct) when asked to explicitly recall which faces provided valid gaze cues and which were invalid. However, these successes should be interpreted cautiously as the same number of participants perform significantly *below* chance (four or less out of 16).[Fig-anchor fig6]

Across all participants, average recall was close to 50% (*M*_prop_ = 0.51, *SD* = 0.50) and a binomial test indicated that the proportion of accurate recall was not significantly above chance (*p* = .341). A Bayesian binomial test found strong evidence for the null hypothesis (BF_01_ = 15.78: pBIC(H1|D) = 0.06 and pBIC(H0|D) = 0.94).

### Discussion

The results of Experiment 2 show that even immediately following gaze cueing, explicit memory for the gaze behavior of individual faces is poor, and the majority of participants perform at chance performance. This is despite the fact that faces are 100% consistent in their behavior (they always provide valid or invalid cues whenever they appear throughout the experiment). There was a gaze-cueing effect consistent with previous studies that show trust learning (Strachan et al., 2016, 2017; Strachan & Tipper, 2017; and Experiment 1 of the current study), and the awareness check procedure happened immediately after gaze cueing had finished.

There is a potential caveat with these results, which is that participants might have interpreted the explicit instruction to ignore the face as a demand of the experimenter, and so these results could reflect demand characteristics as participants underperform at the explicit categorization task. Taking this to its logical extreme, participants would have explicit knowledge of the gaze contingencies, and trustworthiness ratings in other experiments would therefore reflect a judgment made on the basis of explicit knowledge. It is not clear, then, why there would be demand characteristics (participants showing no sensitivity to gaze behavior) in the explicit judgment task and not on the basis of trustworthiness ratings.

The fact that participants perform so poorly at remembering gaze behavior of faces at the point where previous experiments show they can reliably discriminate those faces in terms of trust supports the view that incidental trust learning is an implicit learning effect.

## General Discussion

This study reports the results of two experiments that investigate incidental learning of trust from gaze cues. Experiment 1 found that sleep did not show any benefit for trust learning, either immediately after the sleep interval or following the course of a week. This suggests that inferential trust learning occurs immediately (i.e., during gaze cueing) and does not benefit any further from sleep. Experiment 2 found that explicit recall of gaze behavior immediately after learning was very poor, consistent with the view that memories for specific behavior episodes are lost as trust inferences emerge.

Taken together, the results of these two experiments suggest that people infer trust from gaze cues during an online interaction, and once those trust inferences exist they are independent of the original trustworthy or untrustworthy gaze behavior (i.e., incidental learning of trust from gaze cues does not rely on explicit episodic recall of the gaze behavior). Our data also suggests that once a trust inference is made, targeted recall and rehearsal of the original event does not strengthen the representation any further or protect it from decay. Interestingly, some previous research has suggested that this incidental and implicit trust learning is more consistent in response to invalid faces (Strachan et al., 2016, 2017; Strachan & Tipper, 2017), which means that trust learning mechanisms might prioritize the storage of information about cheating or deception, rather than more general monitoring of trustworthiness (Bell, Buchner, & Musch, 2010; Buchner, Bell, Mehl, & Musch, 2009). A similar pattern of more robust and stable memories of untrustworthy faces was also observed in this study (see Figure 3).

Representing social judgments independent of their origin may be an efficient way of storing information, particularly for unfamiliar people. Rather than explicitly remembering each encounter with an individual, we instead parse each episode to a coarse metarepresentation that accumulates information relevant to future interactions but does not have a trace back to the original episode. Indeed, there is evidence that more abstract/gist embodied states of emotion facilitate the learning and representation of trust. For example, Manssuer et al. (2016) showed that only people who expressed emotional responses during cueing (via EMG recording of facial muscles) later showed learning of trust. Storing information in this way would not only be more efficient, but it would also be less susceptible to decay over time—resulting in the durable effects reported in previous work (Strachan & Tipper, 2017) and here in Experiment 1. The existence of such an adaptive social storage mechanism would furthermore explain why social inferences in the current study were not subject to the same benefits of sleep as hierarchical inferences as observed in previous work (Ellenbogen et al., 2007). If such a mechanism exists, future research could look at modeling it in a computational framework.

Therefore, it appears that learning of trust might have a different time-course to other learning, such as learning the meaning of new words. The former seems to be immediate, whereas the latter word learning is slower and benefits from sleep-dependent consolidation processes. Such contrasts emerge when considering the functions of such learning, where trust would require relatively fast and immediate learning. For example, unlike situations such as word learning where consolidation over hours and days will be sufficient, trust gist has to computed relatively rapidly online during the social interaction, to avoid deception and costly decision making. Hence, the immediacy of trust learning may well contrast to other forms of less costly learning.

An interesting question for future research would be to investigate this mechanism in terms of how explicit awareness and trustworthiness judgments interact and how this might relate to the interaction between embodied emotional reactions and trust learning (Manssuer et al., 2016). We did not look at both trustworthiness judgments and explicit recall in Experiment 2 as there was the possibility of participants using their answers on one measure to inform the other (e.g., reporting that those they distrusted must have looked in the wrong direction). However, if incidental trust learning is an effort-saving mechanism to avoid having to remember the specific gaze behaviors of faces, then an open question is whether there is a reason that a small minority of participants *do* show above-change memory for the faces’ behavior. One prediction might be that those who explicitly remember faces’ gaze behavior would be less likely to show a typical trust effect, because the learned trust effect is an adaptive mechanism to limit the need for this explicit memory.

The results of Experiment 2 also illustrate the importance of investigating this incidental trust learning through different behavioral measures. Where previous studies have predominately used trustworthiness ratings (Manssuer et al., 2015, 2016; Strachan et al., 2016, 2017; Strachan & Tipper, 2017) or decisions in economic games as measures (Rogers et al., 2014), this is the first time we have used a test of explicit awareness. Using other behavioral measures would allow us to investigate further features of this learning. For example, although we have shown no evidence that gaze cueing costs diminish over time, which indicates that participants do not use what they learn about faces to inhibit their reflexive attention shifts to invalid cues (cf. RT analysis of Experiment 1, Supplementary Figure 1), it could be that forming these trustworthiness representations and integrating them into anticipatory forward models that reduce sensitivity to misleading cues are separate processes with different time courses. Indeed, given that sleep has been shown to enhance the formation of relational and associative networks (Cai, Mednick, Harrison, Kanady, & Mednick, 2009; Ellenbogen et al., 2007; Fischer, Drosopoulos, Tsen, & Born, 2006; Wagner, Gais, Haider, Verleger, & Born, 2004), it could be that consolidation affects the integration of learned trust information into anticipatory mechanisms rather than explicit reports of this trust representation as we describe here—that is, you may be able to learn that a person is untrustworthy, but in order to use this information to strategically inhibit the automatic reorienting of attention to their misleading gaze cues you may need to have a period of consolidation. If this is the role that consolidation plays in incidental trust learning, an exciting avenue for future research could be to include a period of consolidation followed by another gaze-cueing session to see if, after having had the chance to consolidate the learned information about the untrustworthiness of invalid faces, people are able to use this strategically to inhibit the automatic reorienting of attention to these cues. Such measures could focus on either RT-based cueing effects as we report here or may supplement this with eye-tracking to detect potentially subtle but dynamic effects.

The current study aimed to explore the role that sleep may play in consolidating incidentally learned trust impressions from gaze cueing behavior. We found that incidental trust learning was remarkably stable over time, even surviving up to a week after the experiment. However, a short nap immediately following the learning phase did not appear to have any effect on participants’ trustworthiness judgments. A second experiment found that participants were not able to explicitly recall the gaze behavior of individual faces even immediately after gaze cueing had ended. This lack of sleep effect and explicit awareness suggests that trust learning involves the formation of a cost-saving gist representation during learning that exists independent of the trust-diagnostic behavior that generated it. Trust representations may be a heuristic metarepresentation of others’ behaviors that allows for efficient, stable, dynamic, and predictive representations for unfamiliar individuals even with minimal information.

## Supplementary Material

10.1037/xlm0000752.supp

## Figures and Tables

**Table 1 tbl1:** Mean Percent Correct (± Within-Subjects Standard Error) for Kitchen or Garage Object Responses in All Four Experimental Conditions in Experiment 1

Group	Valid	Invalid
Sleep	98.02 (±.39)	97.52 (±.43)
Wake	96.35 (±.52)	96.78 (±.49)

## Footnotes

1 This baseline correction (comparing trust ratings in later sessions with the preexperiment rating) allows us to generate a metric of trust *change*, which is the phenomenon that we look to investigate. However, it is worth noting the results of this analysis on the raw trustworthiness ratings without baseline correction: A 2 × 2 ANOVA finds no main effect of session, *F*(2.55,114.96) = 0.08, *p* = .923, $\eta_{p}^{2}$ = 0.00), but did find a main effect of validity, *F*(1,45) = 15.50, *p* < .001, $\eta_{p}^{2}$ = 0.26) and an interaction between the two, *F*(2,90) = 8.21, *p* < .001, $\eta_{p}^{2}$ = 0.15). Follow-up *t*-tests found ratings to valid and invalid faces differed significantly immediately following trust learning, t(45) = 4.27, 95% CI [13.33, 37.10] *p* < .001, *d* = 0.63, following a 90-min interval, *t*(45) = 3.61, 95% CI [8.40, 29.67], *p* < .001, *d* = 0.53, and 1 week later, t(45) = 3.22, 95% CI [4.98, 21.69], *p* = .002, *d* = 0.47.

2 We collected polysomnographic sleep data from participants in the sleep group, which included the length of time spent in REM sleep, N1, N2, and N3 stages, as well as total sleep time. For the sake of completeness, we examined whether time spent in any of these sleep stages correlated with the magnitude of the trust effect (across TMR) at the postinterval or the 1-week sessions. There were no significant correlations with any sleep stages (REM: *r* = .27, *r* = .11; N1: *r* = −.06, *r* = −.02; N2: *r* = .05, *r* = .05; N3: *r* = −.13, *r* = .15) or total sleep time (*r* = .01, *r* = −.11; all *p*s > .221). This was also true if we looked at time in each sleep stage as a percentage of total sleep time (REM: *r* = .27, *r* = .12; N1: *r* = .03, *r* = .07; N2: *r* = −.02, *r* = −.00; N3: *r* = −.13, *r* = .12; all *p*s > .208).

3 Experiment 2 closely matches previously published experiments that have shown reliable incidental trust learning from gaze cues using only five experimental blocks, and which use neutral faces (Strachan et al., 2016, 2017; Strachan & Tipper, 2017). As such, while this design is different from Experiment 1 we are nonetheless confident that this design would produce an incidental trust learning effect if we were able to measure one.
