# Supplementary material for: Investigating the Formation and Consolidation of Incidentally Learned Trust
Source: J Exp Psychol Learn Mem Cogn. 2019 Jul 29;46(4):684–98. doi: 10.1037/xlm0000752 (PMC7115124; doi:10.1037/xlm0000752)
Supplement: Supplementary file 1 [file XLM-2018-0518_Strachan.docx]

# Supplementary Material

## Supplementary Figure 1.


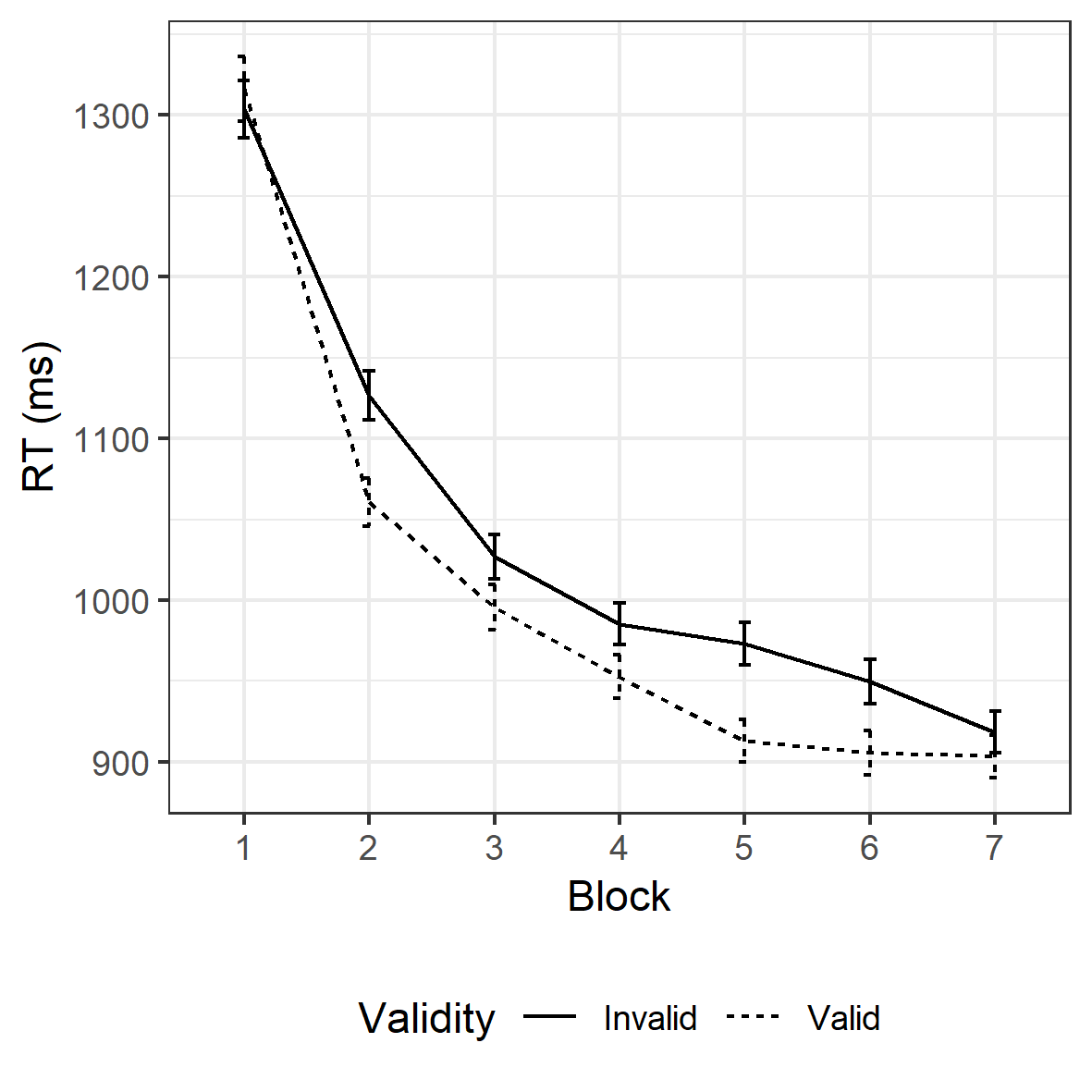


Supp. Figure 1. *Average reaction times across seven blocks to valid (dashed) and invalid trials (solid line). Error bars show ±1 within-subjects standard error.*
